# Influence of Transition Metal Ion Contaminants on the Performance of Amine-Based Solid Sorbents in Direct Air Capture

**DOI:** 10.1021/acs.est.5c11392

**Published:** 2026-05-21

**Authors:** Botagoz Kuspangaliyeva, Ryan P. Lively, Christopher W. Jones

**Affiliations:** School of Chemical & Biomolecular Engineering, 1372 Georgia Institute of Technology, Atlanta, Georgia 30332, United States

**Keywords:** DAC, CO_2_ capture, degradation, oxidation, radical, impurities

## Abstract

Amine-functionalized solid sorbents are a class of sorbent materials proposed for direct air capture (DAC) of CO_2_, yet their long-term performance is susceptible to degradation under realistic operating conditions. Many amines are not thermodynamically stable in air, and amine sorbents oxidize while in use during DAC temperature swing adsorption processes. In this study, we investigate the role of transition metal ion contaminants, specifically Cu^2+^, Fe^2+^, and Ni^2+^, on the oxidative degradation of poly­(ethylenimine) (PEI)-impregnated SBA-15 sorbents. By introducing metal ions via different modes mimicking both synthesis-related impurities and impurities derived from environmental exposure, we systematically evaluate sorbent stability after exposure to dry air at an elevated temperature. Thermogravimetric CO_2_ uptake measurements reveal that even trace levels of Cu and Fe (as low as ∼4 ppm) can lead to measurable sorbent deactivation after oxidative aging, despite negligible loss in the performance of the control samples. *In situ* infrared, UV–vis, and X-ray photoelectron spectroscopies indicate that these metals catalyze radical-driven oxidation pathways, altering the chemical structure of the sorbent and accelerating degradation. Our findings underscore the need to account for trace metal contamination during DAC sorbent synthesis and deployment and highlight the importance of environmental contamination pathways.

## Graphical abstract

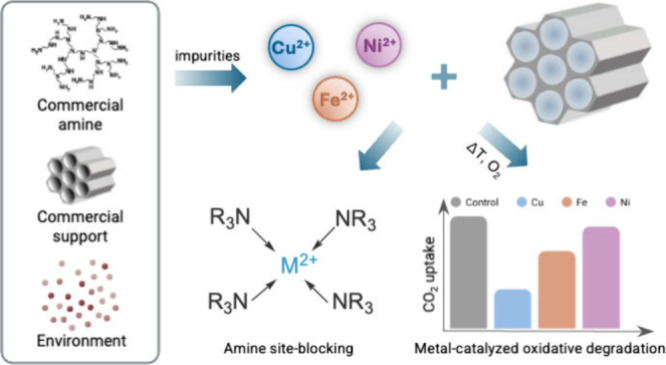

## 1 Introduction

The increasing concentration of atmospheric CO_2_ is a significant driver of climate change, necessitating the development of effective carbon capture technologies. Direct air capture (DAC) stands out as one of the most scalable and promising technological strategies among the various negative emission technologies that capture CO_2_ directly from the ambient air.^1^ Given the ultradilute concentration of CO_2_ in the atmosphere (∼420 ppm), chemisorbent materials capable of forming strong bonds with CO_2_ were found to be effective.^2–4^ Among these chemisorbents, amine-based solid sorbents, particularly poly­(ethylenimine) (PEI)-based sorbents, are commonly used due to their high density of amine groups and demonstrated efficacy under temperature swing adsorption (TSA) or vacuum swing adsorption (VSA) conditions.^5,6^ In terms of scalability and practical deployment, this aminopolymer is commercially available, cost-effective, and easy to handle. Commercial DAC developers also employ amine-functionalized solid sorbents. For example, Zero Carbon Systems (formerly Global Thermostat), Climeworks, Octavia Carbon, and others use amine-based sorbents in their process units.^7,8^ Specifically within the broader class of amine sorbents, branched PEI (MW = 800 Da) has been extensively utilized and supported onto porous oxide materials across the literature for CO_2_ capture from air, flue gas and other mixtures, given its good balance of capacity, stability, kinetics, and availability.

While PEI-based sorbents demonstrate promising performance, their tendency for thermal or oxidative degradation reduces their CO_2_ sorption efficiency over time, presenting a considerable drawback for this material choice.^9–15^ In the context of CO_2_ capture, the DAC adsorbents must contend with an oxygen-rich ambient air environment (21% O_2_) and the temperature swings (up to ∼100 °C) required for CO_2_ desorption during the regeneration cycle. Similar to many commercial organic and polymeric materials, PEI undergoes gradual oxidation even under ambient storage conditions. Sorbents are continually exposed to oxygen not only during DAC cycles but also throughout manufacturing, shipping, storage, and handling processes.^16^ For DAC to be economically advantaged, practical applications expect the adsorbents to maintain stability across tens of thousands of repeated CO_2_ adsorption and desorption cycles.^17^ However, the thermal or oxidative degradation of amines can shorten the sorbent lifetime and increase maintenance and operational costs by necessitating frequent sorbent replacements. Therefore, developing a thorough understanding of the oxidation mechanisms of amines is essential to facilitate their commercial use. Most studies in this area have focused on the effects of temperature and oxygen on the oxidative degradation of sorbents,^9,16,18–22^ with more recent research exploring the role of water and CO_2_.^13–15,23–26^

Most studies on amine-based DAC adsorbents have been conducted under ideal conditions using clean feed streams (including some or all of CO_2_, N_2_, O_2_, and H_2_O), often neglecting the impact of real-world impurities that may strongly interact with PEI, affecting its performance and structural integrity. For practical and scalable DAC applications, it is important to account for ubiquitous environmental contaminants. In this work, we focus on transition metal ions. In aqueous amine solutions, oxidative degradation is well-documented to be catalyzed by redox-active metal impurities, such as Fe^3+^/Fe^2+^ and Cu^2+^/Cu^+^, which generate reactive radical intermediates.^27,28^ Prior liquid amine degradation studies, particularly for monoethanolamine (MEA), have further shown that dissolved metal ions accelerate radical formation, promote amine loss, and produce both volatile and nonvolatile degradation products.^29–31^ While reactor corrosion or anticorrosion metal salts are a common source of metal contamination in liquid-phase systems, solid sorbent systems face different contamination pathways. Trace amounts of transition metals, including Cu, Fe, Ni, and Pd, have been detected in commercial amines such as PEI and tetraethylenepentamine (TEPA),^15,32,33^ as well as in commercial supports like alumina,^15^ potentially introduced during synthesis or leached from metal containers used during processing, transport and storage. Most studies have only reported the presence of these pre-existing trace metal impurities, with a few attempting to analyze their effects on oxidative degradation. For example, Yan and Sayari investigated the impact of deliberate metal contamination by first doping the silica support with Fe or Cu before impregnating it with PEI, though they focused on only two metals and a single contamination pathway.^34^ In these limited studies, even small amounts of these metals were consistently associated with increased oxidation rates of supported amines. Beyond precursor material contamination, metals can also be introduced externally through the DAC process itself. For instance, Isenberg and Chuang found that boiler steam used for sorbent regeneration contains elevated levels of Cu, Fe, and Zn, and their study also demonstrated that these metals accelerate amine degradation when introduced through the steam.^35^ Additionally, outdoor DAC installations are exposed to airborne transition metals (Fe and Cu) from mineral dust, which originates from natural storms^36^ or anthropogenic activities such as urbanization and industrial emissions.^37^ These particles, particularly iron-rich dust, can settle on sorbents as dry deposition, be carried as aerosols under humid conditions, or dissolve in rainwater, leading to further degradation and potential clogging of the adsorbents.

Min et al. have shown that adding a metal chelating agent, such as phosphate ion (trisodium phosphate), during the sorbent preparation could reduce metal-induced oxidative degradation of PEI.^32,33^ While these chelating agents can trap metals present in the commercial PEI, they might not completely prevent oxidation, especially if metals are continually introduced from environmental sources. This suggests that chelators might only be effective for trace amounts of metals already present in the sorbent, and it highlights a critical gap between the controlled experimental conditions and the complex reality of DAC deployment, where long-term exposure to contaminants can lead to cumulative damage. Overall, the contamination and catalytic effects of metal species on solid amine sorbents remain largely underexplored. Given that even trace amounts of metal can influence amine oxidation, it is crucial to further investigate these interactions to inform the development of more degradation-resistant DAC sorbents.

From an environmental perspective, accelerated oxidative degradation has implications beyond sorbent performance alone. Reduced sorbent lifetime increases material production and replacement frequency, thereby amplifying associated lifecycle emissions and resource demands. In addition, metal-catalyzed oxidation may promote the formation of volatile nitrogen-containing degradation products (e.g., ammonia or low-molecular-weight amines), which could contribute to secondary air quality impacts. While quantitative environmental assessment lies beyond the scope of this mechanistic study, identifying the intrinsic susceptibility of PEI-based sorbents to trace metal contamination is a necessary step toward evaluating the broader environmental footprint and long-term durability of DAC technologies.

The objective of this work is to establish a mechanistic and comparative understanding of transition metal-accelerated oxidative degradation in PEI-based DAC sorbents under controlled laboratory conditions. This work is not intended to replicate full DAC unit performance or to predict absolute sorbent lifetimes under specific process configurations. Rather, by employing a simplified and well-defined powder system, we intentionally decouple intrinsic chemical degradation pathways from geometric and mass-transfer limitations present in structured contactors, thereby isolating intrinsic material susceptibility. While degradation rates in structured DAC systems and humid environments may differ quantitatively, identifying metal-dependent chemical vulnerability provides a necessary foundation for evaluating degradation risk, material durability, and associated lifecycle implications under practical deployment scenarios.

Here, we systematically explore the influence of Cu, Fe, and Ni ions on the performance and oxidative degradation of PEI supported on mesoporous silica (SBA-15). To simulate real-world exposure scenarios, we investigate three different contamination pathways: mode 1, contamination within the aminopolymer (PEI); mode 2, contamination originating from the support (SBA-15); and mode 3, contamination introduced from environmental sources (aerosol and rain). By comparing these pathways, we aim to assess the impact of metal introduction on sorbent performance and degradation under accelerated aging conditions, while leveraging the first mode for in-depth mechanistic studies due to its uniform metal distribution, which provides a controlled system for fundamental investigations.

## 2 Materials and Methods

### 2.1 Synthesis of SBA-15

Mesoporous SBA-15 silica was synthesized following our previously reported procedure.^38,39^ Briefly, 24 g of Pluronic P-123 block copolymer (a triblock copolymer of poly­(ethylene oxide)–poly­(propylene oxide)–poly­(ethylene oxide), EO_20_PO_70_EO_20_, Sigma-Aldrich) was dissolved in 636 g of deionized (DI) water and 120 mL of 12.1 M HCl, and the mixture was stirred vigorously for 3 h until completely dissolved. Then 46.6 g of tetraethyl orthosilicate (TEOS, Acros Organics) was added dropwise to the mixture and stirred at 40 °C for 20 h, during which a white precipitate formed. The mixture was then heated to 100 °C and maintained at this temperature for 24 h without stirring. Afterward, the reaction was quenched with 400 mL of DI water, and the precipitate was filtered and extensively washed with DI water. The filtered precipitate was dried in an oven at 75 °C for 12 h and then calcined at 550 °C for another 12 h. SBA-15 was selected as a model support because its synthesis from TEOS and P-123 yields a silica framework with very low intrinsic metal content, providing a clean baseline needed for controlled introduction of metals and for isolating the mechanistic effects of contaminants.

### 2.2 Preparation of PEI/SBA-15 Composite

The sorbents were prepared by impregnating SBA-15 with 30 wt % commercial poly­(ethylenimine) (PEI, branched, 800 g/mol LS, Sigma-Aldrich). Before impregnation, the silica support was dried overnight under vacuum (<20 mTorr) at 120 °C to eliminate residual moisture. PEI was first dissolved in methanol (Sigma-Aldrich), and the resulting solution was added to the methanol-dispersed SBA-15. The mixture was stirred at room temperature overnight to facilitate uniform distribution. Methanol was then removed by rotary evaporation at 50 °C. The resulting powder was further dried under vacuum (<20 mTorr) for 3 days at room temperature and then stored in a dry argon environment until use.

### 2.3 Preparation of Contaminated Sorbents

Transition metal ions, copper (Cu), iron (Fe), and nickel (Ni), were selected due to their prevalence as impurities in commercial amines^15,32,33^ and supports,^15^ and their widespread presence in the environment, both naturally and due to anthropogenic activities.^37,40^ Metal ions were introduced in the form of their acetate salts: Cu­(CH_3_COO)_2_ (Tokyo Chemical Industry), Fe­(CH_3_COO)_2_ (Sigma-Aldrich), and Ni­(CH_3_COO)_2_ (Sigma-Aldrich). To simulate different contamination pathways, three distinct incorporation methods were employed during the preparation of contaminated PEI/SBA-15 sorbents, as illustrated in Figure 1. In mode 1, metal ions were directly incorporated into the PEI, simulating contamination during polymer manufacturing or handling, and providing a relatively uniform distribution of metal ions throughout the PEI phase. In mode 2, metal contamination was introduced at the support level before PEI loading, simulating supports with pre-existing contamination from synthesis precursors (e.g., clay-derived alumina). In mode 3, contamination was introduced from simulated environmental sources, mimicking real-world exposure such as aerosols and water droplets.

**Figure 1:**
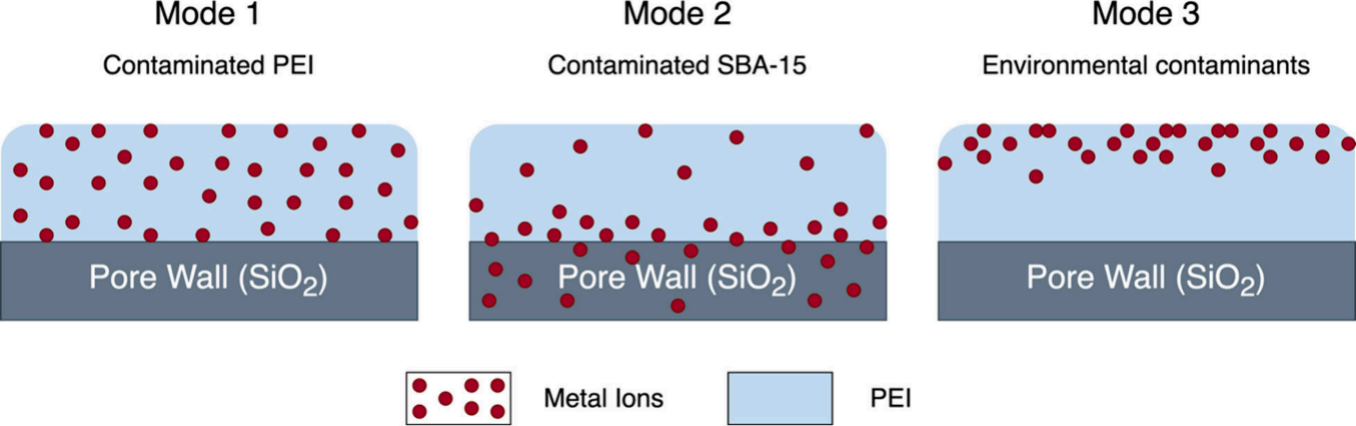
Schematic of expected metal distribution across contamination modes.

#### 2.3.1 Mode 1 (Contaminated PEI)

Metal salts were dissolved in methanol and mixed with PEI to achieve targeted PEI-to-metal molar ratios. The resulting metal-loaded PEI solution was then impregnated onto predried SBA-15 using the method described in section 2.3.

#### 2.3.2 Mode 2 (Contaminated SBA-15)

SBA-15 was mixed with metal salt in methanol, with the metal amount calculated to match the target PEI-to-metal ratio used in other modes. After rotary evaporation, the dried material served as mode 2.1. A portion was further calcined at 550 °C for 6 h to yield mode 2.2. Both the mixed and calcined supports were then impregnated with pristine PEI to form the final composite sorbents.

#### 2.3.3 Mode 3 (Environmental Contaminants)

To simulate real-world exposure, dry PEI/SBA-15 sorbents were contacted with either metal-contaminated aerosols (mode 3.1) or droplets (mode 3.2) in a gently stirred three-neck round-bottom flask. In mode 3.1, a nebulizer delivered metal-laden water aerosols through the central neck at 0.2 mL/min, while the other two necks remained open to ambient air to prevent pressure buildup. In mode 3.2, discrete droplets were added via the central neck at ∼10 min intervals, avoiding slurry formation and allowing gradual water absorption and drying. In both modes, total liquid volume and metal concentrations were adjusted to target the same PEI-to-metal molar ratios as in other experimental modes.

### 2.4 Sorbent Characterization

#### 2.4.1 Nitrogen Physisorption

N_2_ physisorption measurements were conducted on a Micromeritics TriStar II at 77 K using ∼180 mg of the sorbent. Before the analysis, the sample was degassed under a vacuum for 10 h at 90 °C. The pore volume, pore fill, pore size distribution, and BET surface area of the sorbent were then determined based on the obtained physisorption data.

#### 2.4.2 Elemental Analysis

The concentrations of Cu, Fe, and Ni were quantified by inductively coupled plasma mass spectrometry (ICP–MS), while the C, H, and N contents were measured using a combustion-based elemental analyzer. All analyses were performed by Intertek (Whitehouse, NJ).

#### 2.4.3 Thermogravimetric Combustion Analysis

The organic content of the sorbents was quantified using thermogravimetric analysis (TGA, TA Instruments Discovery 550). Samples were first dried under nitrogen at 110 °C for 1 h, then heated to 700 °C in a 90 mL/min flow of zero-grade air (21% O_2_ in N_2_) at a ramp rate of 10 °C/min. The PEI loading was determined from the weight loss between 120 and 700 °C, relative to the combustion profiles of bare or metal-contaminated SBA-15.

#### 2.4.4 CO_2_ Adsorption Capacity

CO_2_ sorption capacities were measured using thermogravimetric analysis (TGA, TA Instruments Q500). Approximately 10 mg of sample was used to ensure uniform powder thickness across all measurements. Samples were pretreated under nitrogen (90 mL/min) at 100 °C for 1 h, then cooled to 30 °C at a rate of 10 °C/min. CO_2_ uptake was evaluated at 30 °C using a gas stream containing 400 ppm of CO_2_ balanced with inert gas, with measurements collected over a 3 h period.

### 2.5 Sorbent Testing

#### 2.5.1 Accelerated Aging Treatment

Accelerated oxidative aging experiments were performed using thermogravimetric analysis (TGA, TA Instruments Discovery 550). Approximately 20 mg of each sorbent was loaded onto a platinum pan. Samples were first pretreated under a nitrogen atmosphere (100 mL/min) at 100 °C for 1 h to remove physisorbed water and CO_2_. The temperature was then increased to 120 °C, and the gas flow was switched to dry air (100 mL/min; 21% O_2_ in N_2_) for 4.5 h to induce oxidative degradation. After aging, the system was returned to nitrogen flow and cooled to 25 °C to prevent further oxidation.

#### 2.5.2 *In Situ* FTIR Spectroscopy

*In situ* diffuse reflectance infrared Fourier transform spectroscopy (DRIFTS) was conducted using a Thermo Scientific Nicolet 8700 IR spectrometer equipped with a Harrick Praying Mantis flow-through cell. The powder sorbent was first loaded into the cell and pretreated under a nitrogen flow (100 mL/min) at 100 °C for 1 h to remove preadsorbed species. Following this, the temperature was increased to 120 °C and stabilized before introducing dry air (21% O_2_ balanced with N_2_) at 100 mL/min to initiate oxidative aging, consistent with the TGA aging protocol. Spectra were collected every 2 min over the 4.5 h aging period. An initial background spectrum was recorded at 120 °C under N_2_ (*t* = 0 h), and all subsequent spectra were referenced to this baseline. After aging, the gas flow was returned to N_2_ for 30 min, and the cell was cooled to ambient temperature. Difference spectra were used to monitor chemical changes in the sorbents over time.

#### 2.5.3 X-ray Photoelectron Spectroscopy (XPS)

XPS measurements were performed using a Thermo Fisher Scientific K-Alpha spectrometer. Data acquisition and peak fitting were conducted using the Thermo Scientific Avantage software. Elemental ratios, including N/Metal and Si/Metal, were calculated to assess the distribution and surface enrichment of metal contaminants in the sorbents.

#### 2.5.4 UV–Vis Spectroscopy

Diffuse reflectance UV–vis spectra were collected using a Varian Cary 5000 spectrophotometer equipped with a Varian Internal Diffuse Reflectance Accessory (DRA) 2500. Air was used as the reference. Measurements were performed on dry powder samples at room temperature over a wavelength range of 200–800 nm, with a scan rate of 600 nm min^–1^. The raw reflectance data were normalized to 100% at 800 nm to facilitate comparison between samples.

## 3 Results and Discussion

### 3.1 CO_2_ Uptake Performance of Metal-Contaminated PEI

We first examined the effect of metal contamination within the amine phase by preparing PEI-functionalized sorbents with Cu, Fe, or Ni preincorporated into the polymer prior to impregnation onto mesoporous SBA-15. Characterization of the control sorbent (PEI/SBA-15) is provided in Figure S1, and a summary of textural properties for all samples is included in Table S1. Overall, the control and metal-contaminated sorbents exhibited comparable surface area, pore volume, and pore filling, suggesting that differences in performance are primarily due to chemical effects rather than textural variations.

As shown in Figure 2, sorbents were evaluated for CO_2_ adsorption capacity before and after oxidative aging at 120 °C in dry air (21% O_2_ in N_2_) for 4.5 h across five different PEI-to-metal molar ratios. The 120 °C condition was adopted as an accelerated aging protocol to enable mechanistic assessment on practical laboratory time scales, as oxidative degradation progresses slowly at typical DAC regeneration temperatures (≤100 °C).^15,41^ To evaluate temperature dependence, additional aging experiments were performed at 80 °C (representing milder thermal conditions) and 160 °C (more aggressive stress-test) for the control sorbent and representative Cu- and Fe-contaminated samples (PEI/metal = 1000:1). While lower temperatures slow degradation and higher temperatures accelerate it, the relative metal-dependent ordering (Cu > Fe ≫ control) is preserved across these conditions (Figure S2a).

**Figure 2:**
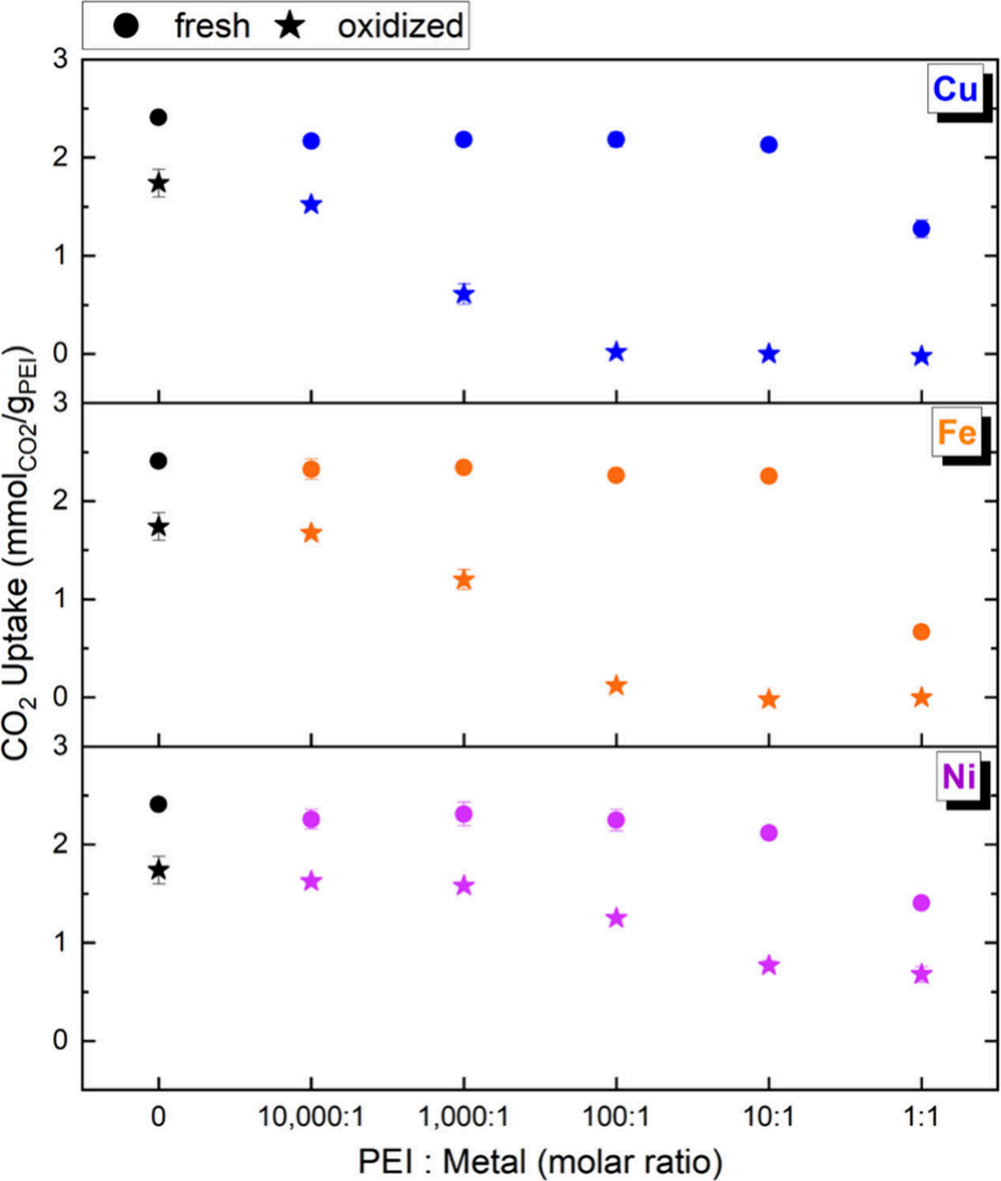
CO_2_ uptake performance of control and metal-contaminated PEI sorbents (Cu, Fe, and Ni) at varying PEI/metal molar ratios before and after oxidative aging at 120 °C for 4.5 h in air (21% O_2_ in N_2_). CO_2_ adsorption was performed using dry 400 ppm of CO_2_ at 30 °C. Each data point represents the mean of *n* = 2 independently prepared sorbents measured separately. Error bars represent ±half of the range between replicates [i.e., (max – min)/2]. In several cases, the error bars are smaller than the marker size and therefore not visible. Corresponding numerical values are provided in Table S2.

To further examine behavior at lower temperature, long-duration aging was also conducted at 80 °C for 20 h and 5 days. Although absolute uptake losses differ due to temperature-dependent kinetics, the same qualitative degradation hierarchy emerges under extended exposure at 80 °C (Figure S2b). This consistency supports the use of 120 °C aging as a comparative screening protocol for relative susceptibility, while further indicating that intrinsic metal-dependent trends persist under lower temperature aging conditions.

In the fresh state, metal-contaminated sorbents showed relatively modest reductions in CO_2_ capacity (up to ∼10%) compared to the control, except at the highest PEI-to-metal molar ratio of 1:1 (Figure 2). At this ratio, uptake dropped by ∼45% for Cu and Ni and ∼70% for Fe. This suggests that direct metal coordination begins to substantially interfere with amine availability and polymer chain mobility only at elevated metal concentrations. Because amines are known to strongly chelate transition metals, an interaction leveraged in heavy metal remediation applications,^42–45^ coordinative site-blocking is likely the dominant effect. Complexation studies report that one metal ion can coordinate with up to four nitrogen atoms of amines in aqueous solution,^46^ and such multidentate interactions can reduce available reactive amines while restricting polymer free volume, thus contributing to CO_2_ diffusion limitations.^35^ Elemental analysis confirmed that the 1:1 PEI/metal samples contain up to 1.8 wt % metal (Table S3), enough to drive extensive binding, possible PEI chain cross-linking, and a measurable drop in CO_2_ uptake. While at low metal levels, the fraction of amine sites that chelate each ion is modest, so most primary and secondary amines remain available for CO_2_ sorption formation, and the chains are not yet completely sterically hindered due to metal coordination.

Following oxidative aging, all metal-contaminated sorbents experienced significantly greater capacity loss compared to the control, with capacity retention decreasing monotonically with metal ion concentration. Cu- and Fe-containing samples showed the most dramatic loss by completely losing CO_2_ capacity at loadings of 100:1 and higher, even though their respective fresh sorbents showed similar uptake capacities as the control. Even at more dilute concentrations (1000:1 PEI to metal), only 25–50% of the initial capacity was retained, whereas the control sorbent preserved 72%. Notably, Cu-contaminated sorbents at just 10 000:1 (∼4 ppm of Cu, Table S3) retained only 63% capacity after oxidative aging, underscoring the significant impact even trace redox metal impurities can have on sorbent stability. Such low-level Cu or Fe contamination, whether introduced during sorbent synthesis or from operational exposure, may substantially reduce sorbent lifetime. These findings are consistent with prior studies reporting metal impurities in commercial amines and supports. For example, Carneiro et al. found up to 12 ppm Fe in commercial γ-Al_2_O_3_,^15^ and Choi et al. reported ∼17 ppm Fe and ∼7 ppm of Cu in commercial PEI (MW 1200).^33^ Min et al. reported even higher levels in TEPA, with 160 ppm Fe, 47 ppm of Cu, and 4 ppm of Ni, noting a trend of higher contamination in lower MW amine polymers.^32^

Ni, in contrast, is much less redox-active under these conditions, and although some site-blocking is observed, oxidative degradation is less extensive. Ni-containing samples consistently retained the highest capacity postaging (∼28% of control at 1:1), reinforcing that the catalytic degradation trend follows Cu > Fe ≫ Ni. This sequence aligns with the redox behavior of these metals, where Cu^2+^/Cu^+^ and Fe^3+^/ Fe^2+^ readily cycle to generate radicals in the presence of O_2_ and H_2_O,^47^ and can directly oxidize amines to form amine radicals via abstracting an electron from the nitrogen.^28^ In contrast, Ni^2+^ is kinetically sluggish because the Ni^2+^/Ni^3+^ couple has a relatively low standard reduction potential. Visual evidence of degradation is shown in Figure S3, where the pristine white sorbent turned pale yellow (indicative of mild oxidation), while the Cu-contaminated blue sorbent darkened to brown after oxidative aging, consistent with more severe oxidative decomposition. We suggest a dual performance-loss mechanism: (i) loss of CO_2_ uptake capacity via metal-induced amine site blocking and (ii) metal-catalyzed oxidative degradation under aerobic thermal conditions. The spectroscopic evidence for these degradation pathways is discussed in the Infrared and UV–vis spectroscopy section.

### 3.2 Effect of Metal Contamination Pathway on Sorbent Stability

To evaluate how different contamination routes influence sorbent performance, we compared CO_2_ uptake across five metal incorporation modes at a fixed PEI-to-metal molar ratio of 1:1 (Figure 3). These pathways are defined as follows: mode 1, metal-contaminated PEI (mimicking commercial polymer impurities); mode 2.1, metal introduced by physical mixing with the support (uncalcined); mode 2.2, metal incorporated into the support and calcined before PEI loading; mode 3.1, metal deposited via aerosol; and mode 3.2, metal introduced through aqueous droplets. While detailed synthesis protocols and the hypothesized metal distributions are described in the Experimental section, we highlight that calcination in mode 2.2 may anchor metal ions more firmly to the silica surface. This mimics contamination scenarios observed in commercial supports such as γ-alumina, which often contains residual transition metals from its clay- or bauxite-derived precursors.^48–50^ Recent work by Carneiro et al. showed that support composition and native metal impurities significantly affect PEI oxidation.^15^ Modes 3.1 and 3.2 simulate environmental exposures relevant to outdoor DAC systems. Notably, mode 3.1 samples exhibited substantially lower final metal concentrations due to losses from aerosolization, whereas all other modes were prepared to achieve a nominal 1:1 PEI/metal molar ratio (Table S4). This design allows a systematic comparison of how metal origin, within the amine (mode 1), the support (modes 2.1 and 2.2), or the environment (modes 3.1 and 3.2), influences sorbent behavior.

**Figure 3:**
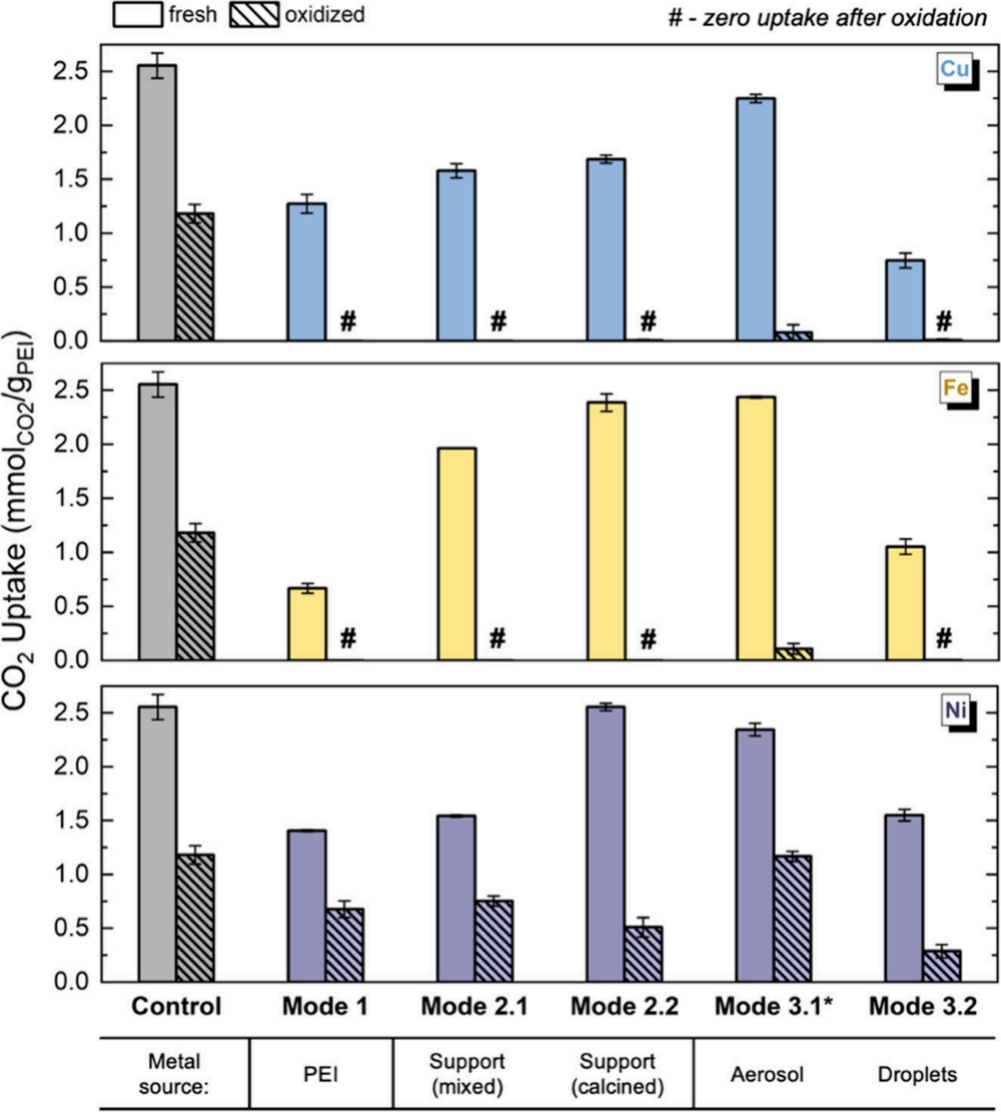
CO_2_ uptake capacities of the control sorbent and samples contaminated with Cu, Fe, or Ni through five pathways, each prepared at a PEI/metal molar ratio of 1:1. Adsorption measurements were performed before and after oxidative aging at 120 °C for 4.5 h in air (21% O_2_ in N_2_), using dry 400 ppm of CO_2_ at 30 °C. The asterisk (∗) symbol indicates that mode 3.1 samples had reduced metal loading due to aerosol loss (see Table S4). The hash (#) symbol denotes samples with zero residual capacity after aging. Each data point represents the mean of *n* = 2 independently prepared sorbents measured separately. Error bars represent ±half of the range between replicates [i.e., (max – min)/2].

For Fe, fresh CO_2_ uptake in modes 2.1 and 2.2 was approximately three times higher than in mode 1, approaching the performance of the pristine control. This suggests that when Fe is introduced via the support, it preferentially associates with the silica surface rather than the PEI phase. A similar observation was reported by Yan and Sayari,^34^ who found that preloading Davisil silica with 1000 ppm Fe did not compromise first-cycle CO_2_ uptake. In contrast, Ni-contaminated sorbents in mode 2.1 performed similarly to mode 1, likely because Ni^2+^ acetates can still diffuse into the polymer and coordinate with amines. However, mode 2.2 showed a marked improvement for Ni, with uptake approaching the control. This may be attributed to the calcination step converting Ni­(OAc)_2_ to NiO, anchoring the metal closer to the silica interface and minimizing amine-metal contact. Unlike Fe and Ni, Cu-contaminated samples showed comparable performance losses across modes 1, 2.1, and 2.2. Even when preloaded onto the support and calcined, Cu appears to migrate into the PEI phase and coordinate with amine groups, resulting in mode-independent amine deactivation. This behavior is consistent with the Irving-Williams series, which ranks metal–ligand stability as Cu^2+^ > Ni^2+^ > Fe^2+^ (from “soft” to “hard” metals), indicating Cu’s stronger affinity for nitrogen donors (e.g., PEI) and Fe’s relative preference for oxygen-based ligands (e.g., silanol).^51^

Mode 3.1 samples contained substantially lower metal concentrations, approximately 25 times lower for Cu, 40 times for Ni, and 160 times for Fe, compared to the intended 1:1 molar ratio (1 metal per ∼20 N; Table S4), due to losses during aerosol delivery. Consequently, fresh CO_2_ uptake remained relatively high for all three metals and followed the same trend observed in dilute mode 1 samples in Figure 2 (Cu > Ni > Fe). However, after oxidation, the Cu- and Fe-contaminated samples in mode 3.1 exhibited near-zero CO_2_ capacity, illustrating that even trace (ppm-level) contamination can critically impact long-term sorbent performance. In mode 3.2, Cu-contaminated samples exhibited notably reduced fresh uptake, likely because solvated metal ions blocked surface-accessible amine sites, thereby limiting CO_2_ diffusion into the PEI film. Interestingly, Fe-contaminated sorbents in mode 3.2 showed higher uptake than their mode 1 counterpart. We hypothesize that this may be due to partial oxidation of Fe^2+^ to Fe^3+^ (e.g., FeOOH formation) in the presence of water and oxygen. The color of the fresh sorbent included more of an orange hue compared to other modes, supporting this hypothesis. In contrast, mode 1 samples were prepared in methanol, where Fe^2+^ remains more stable due to rapid PEI chelation. For Ni, the fresh uptake in mode 3.2 was similar to modes 1 and 2.1, reaffirming that only the calcined condition (mode 2.2) or lower metal loading (mode 3.1) allowed the retention of high performance.

As illustrated in Figure 1, we hypothesized that metal ions occupy different locations within the sorbent depending on the contamination pathway: in mode 1, metals are expected to be relatively uniformly distributed within the PEI; in mode 2, metals are more likely concentrated near the support interface, particularly in the calcined cases (e.g., Fe and Ni); and in mode 3, metals are presumed to remain primarily on or near the outer surface of the PEI due to environmental deposition. To explore whether metal location affects the fresh CO_2_ uptake trends, we compared metal-to-nitrogen molar ratios from both bulk elemental analysis (EA) and surface-sensitive XPS for selected samples across the contamination modes (Table S4). In mode 1, Cu and Ni exhibited similar EA and XPS ratios (Cu and Ni: 0.05 vs 0.05), suggesting uniform metal dispersion throughout the PEI. Fe, however, showed a notably higher XPS ratio (0.09) than its bulk value (0.05), indicating surface enrichment of Fe on the PEI. In mode 2.1, Cu appeared modestly more buried (0.03 XPS vs 0.05 EA), while Fe again showed surface localization on the PEI (0.09 XPS vs 0.04 EA), more pronounced than in mode 1. Notably, although both modes 1 and 2.1 showed similar Fe surface enrichment, the fresh sample prepared via mode 2.1 exhibited ∼3× higher CO_2_ uptake than mode 1. This suggests that surface Fe presence alone does not fully dictate fresh-state performance and that factors such as local Fe coordination environment or interactions with the support may play significant roles. In mode 2.2, XPS detected no Fe on the PEI surface, confirming strong anchoring or burial within the support after calcination. In mode 3.2, Fe remained enriched at the PEI surface (0.11 XPS vs 0.05 EA), consistent with our hypothesis for environmentally contaminated sorbents. Collectively, these data suggest that Fe is typically found at or near the PEI surface unless strongly anchored via calcination, while Cu and Ni more closely follow the hypothesized spatial trends across modes.

Interestingly, despite variations in fresh-state performance, oxidized samples exhibited similar CO_2_ uptake across all modes (Figure 3). This suggests that high-temperature oxidative aging may promote PEI chain relaxation and metal ion mobility, ultimately redistributing metals and evening out performance differences regardless of how metal ions get into the sample. To further explore metal mobility, we analyzed Metal/Si molar ratios from surface-sensitive XPS for some samples before and after oxidation (Table S5). Across most modes, clear changes in Metal/Si ratios were observed between fresh and aged sorbents, indicating significant metal redistribution during accelerated oxidative aging. Overall, these results emphasize the mobility of metal ions under aging conditions, suggesting that as they travel within the PEI, they can further interact with and deactivate PEI, contributing to the convergence in CO_2_ uptake performance among oxidized samples.

Finally, CO_2_ uptake measurements at a lower PEI/metal ratio of 100:1 (Figure S4) reveal the same trend: fresh uptake in mode 2.1 was slightly higher than in mode 1 for all metals, but oxidation again led to similarly diminished performance after oxidation. Although only two contamination modes were tested at this concentration, these data reinforce that contamination pathway impacts fresh-state performance, but thermal treatment largely overrides these distinctions in the long term.

### 3.3 Infrared and UV–Vis Spectroscopy

Diffuse reflectance infrared Fourier transform spectroscopy (DRIFTS) analysis of the activated sorbents revealed that metal ion incorporation subtly perturbs the amine environment without altering the primary chemical structure of PEI (Figure S5). All samples exhibited characteristic PEI peaks, including N–H stretching bands at ∼3360 and ∼3300 cm^–1^, C–H stretching at ∼2940 and 2820 cm^–1^, N–H bending near 1600 cm^–1^, and C–H bending around 1460 cm^–1^. Upon metal addition, the N–H bending peak showed noticeable shifts and broadening relative to the control, suggesting coordination between amine groups and metal ions. While the C–H stretching region remained largely unchanged, the C–H bending peak became less intense in the metal-containing samples, indicating that metal coordination may induce conformational changes or structural disorder within the PEI backbone. These effects are consistent with literature reports of metal-amine coordination, where vibrational bands shift or change intensity due to bonding between metal ions and nitrogen atoms.

To track the evolution of surface species during oxidative aging, *in situ* DRIFTS spectra were collected under dry air (21% O_2_ in N_2_) at 120 °C for 4.5 h, mirroring the aging conditions used in previous sections. Time-resolved difference spectra were obtained by subtracting the spectrum at *t* = 0 min of aging from those at various time points (up to 270 min). In Figure 4, we note the distinguishable 1664–1687 and 1604–1607 cm^–1^ bands formed during the oxidative aging of the control and metal-contaminated PEI sorbents with Cu, Fe, and Ni. The first spectral region at around 1675 cm^–1^ corresponds to the formation of carbonyl (C=O) and/or imine (C=N) species on the backbone of PEI with the course of the oxidative aging, which has been discussed in literature as a characteristic of amine-related oxidation products and serves as a proxy for sorbent deactivation.^9,12,20,52,53^ The latter spectral region around 1605 cm^–1^ overlaps with the peaks associated with the N–H bending (primary amine deformation) mode of amines.^11,14,15,54,55^

**Figure 4:**
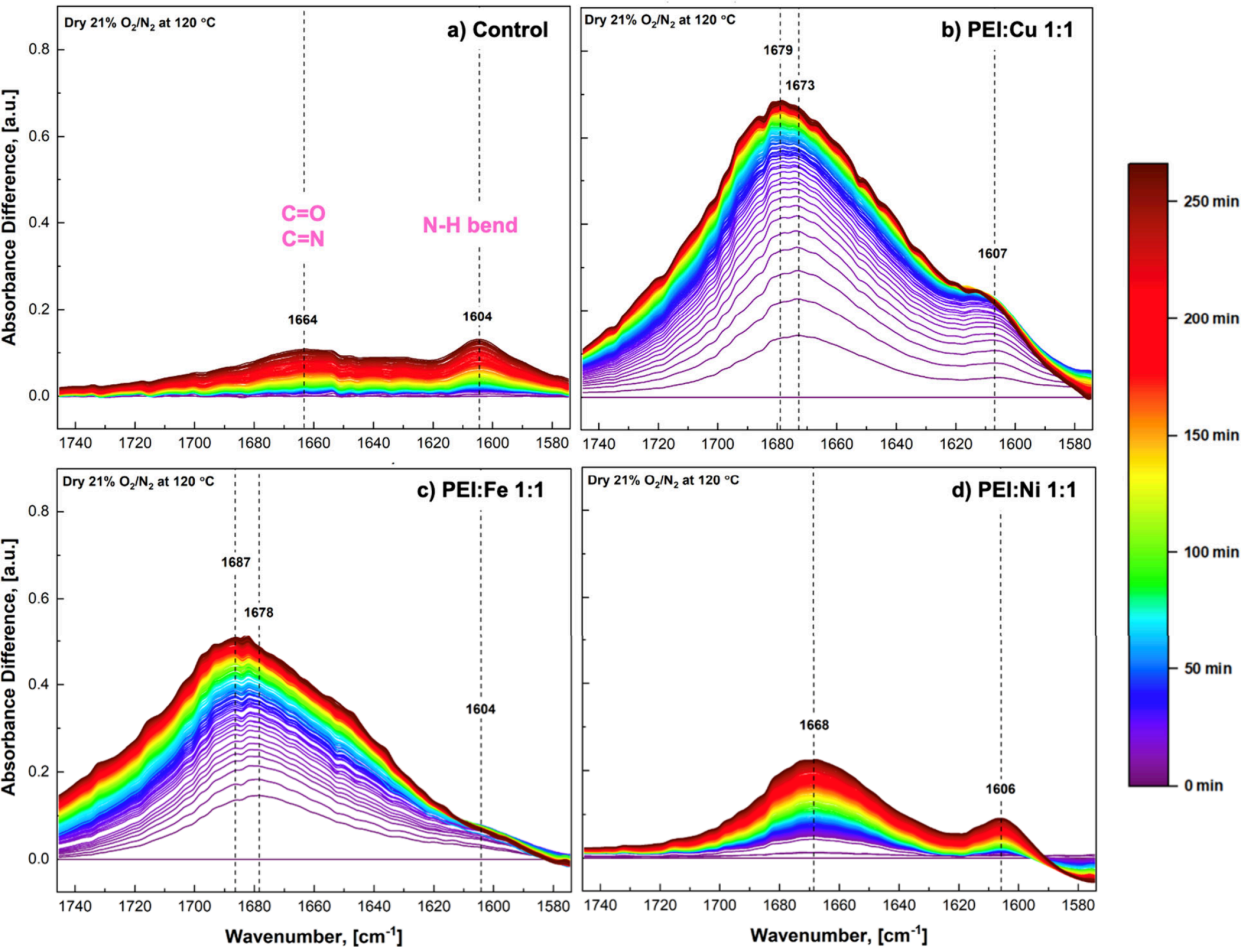
Difference DRIFTS spectra collected during *in situ* oxidative aging of (a) control and (b–d) metal-contaminated PEI sorbents (Cu, Fe, and Ni) under 21% O_2_ in N_2_ at 120 °C for 4.5 h. All contaminated samples were prepared at a PEI/metal molar ratio of 1:1.

Radical-driven autoxidation is central to understanding the metal-accelerated degradation observed in this study. Building on earlier studies of amine oxidation,^10,16,18,20,28,56–58^ our group has recently proposed a detailed C–N bond cleavage mechanism during PEI oxidative degradation, resulting in carbonyl/imine species, newly formed primary amines, and other low molecular weight amine products.^15^ Under dry conditions, degradation begins with the radical initiation step, namely by amino alkyl radicals formation along the PEI chain due to thermal stress,^59^ which leads to chain relaxation and breakage, and/or due to an already present ppm amount of metal impurities within the sorbent that catalyzes free radical formation (see schematic in Figure S6).^15,33,34^ When exposed to air, these radicals react with O_2_, forming in-chain peroxyl radicals (ROO^•^) that further abstract hydrogen from the nearby PEI chain, generating hydroperoxide (ROOH) intermediates.^60,61^ The breakdown of ROOH groups leads to carbonyl compounds C=O within the PEI chain through terminal C–N bond cleavage. Depending on the location of the scission (primary, or secondary amines), new primary amines, imine species, and volatile products (e.g., ammonia and 2-aminoacetaldehyde) can form.^15,16^

Moreover, another recent combined experimental and first-principles simulation study revealed an alternative degradation pathway under CO_2_-free conditions involving C–C bond cleavage.^62^ While following the same initial radical-driven steps, the decomposition of ROOH intermediates can yield amide products containing C=O groups and alkyl radicals through a nearly barrierless C–C bond scission. In contrast, C–N bond cleavage exhibits a relatively high activation energy under a CO_2_-free atmosphere.^62^ A conceptual schematic integrating previously reported radical-driven pathways with the hypothesized role of metal redox cycling is provided in Figure S6. This illustration integrates the C–N and C–C bond-cleavage routes with the reactive oxygen species (ROS)-mediated activation steps attributed to transition metal contaminants. Overall, oxidative degradation (autoxidation) of amines is a complex process associated with the occurrence of free-radical and chain scission reactions taking place simultaneously in distinct regions of the polymer backbone.^63^ These proposed mechanisms explain why amine oxidation causes CO_2_ capacity loss via (1) conversion of basic amines to inactive carbonyl/imine species and (2) overall amine loss due to polymer fragmentation.

The increased intensity of the N–H bending (∼1605 cm^–1^) has been reported in our recent work as the formation of new primary amine species as a result of the C–N bond scission at secondary amine sites.^15^ Using this assignment, the generation of new primary amines with time is especially pronounced in the Cu-contaminated sample (Figure 4b), indicating increased C–N bond cleavage. This observation aligns with the severe CO_2_ capacity loss seen for Cu samples after oxidation compared to other metal ions (Figure 2). In contrast, the N–H band is less pronounced in the Fe-contaminated sample (mode 1; Figure 4c), though more apparent in spectra from Fe-loaded supports (modes 2.1 and 2.2; Figure S7). This may be attributed to the low initial CO_2_ uptake observed for the fresh mode 1 Fe sample (Figure 3), indicating that a significant fraction of the amines may have already been inaccessible or deactivated by Fe in the fresh state. As a result, fewer secondary amines were available for oxidative C–N bond cleavage during aging, leading to a less pronounced formation of new primary amines. The intensity of the 1675 cm^–1^ C=O/C=N band follows the trend Cu > Fe ≫ Ni, matching the order of degradation severity observed in Figures 2 and 3. More active catalysts like Cu and Fe in PEI accelerate carbonyl/imine formation more rapidly than primary amine generation, particularly within the first 30 min of aging.

Figure S8 expands the spectral view to 1000–3500 cm^–1^, highlighting two prominent negative peaks at ∼2900 and ∼2800 cm^–1^ across all samples. These features correspond to C–H stretching vibrations and are indicative of aliphatic (−CH_2_−) content loss due to polymer backbone degradation. The C–H depletion is notably more pronounced in Cu- and Fe-contaminated sorbents, likely resulting from hydrogen atom abstraction from the −CH_2_– groups, a key step in initiating radical-driven chain scission via C–N or C–C bond breakage. In contrast, the control sample exhibits only minor changes in the C–H, C=O/C=N, and N–H bending regions (Figure S8a), consistent with slower oxidative degradation, as reflected by a PEI weight loss of 3.9% during oxidation in TGA. Metal identity clearly influences oxidation kinetics, since Cu and Fe dramatically amplify carbonyl/imine formation and C–H loss (Figures S8b and c), corroborated by their higher polymer weight losses during aging (25.8% for Cu and 24.2% for Fe). This implies that oxidation can partly cleave the polymer backbone into smaller fragments, which can be thermally evaporated. In these samples, the emergence of new N–H stretching bands in the 3300–3400 cm^–1^ range signals the formation of new amine environments, while concurrent negative C–H bands confirm backbone cleavage, both consistent with the observed declines in CO_2_ capacity and weight loss. Ni, by contrast, shows only modest changes and retains more PEI (12.1% loss) (Figure S8d), consistent with its lower redox activity under these conditions.

Collectively, the more intense growth of C=O/C=N and primary amine bands in metal-contaminated samples supports the conclusion that metals accelerate PEI autoxidation by lowering the activation barrier required for the radical initiation step.^64^ This is likely mediated by ROS, such as superoxide or hydroxyl radicals, generated via metal and O_2_ interactions,^65,66^ producing alkyl radicals that readily react with triplet O_2_ to propagate autoxidation.^62,64^ Notably, Cu^2+^/Cu^+^ and Fe^2+^/Fe^3+^, can drive Fenton-type redox cycles, forming *in situ* hydroxyl and superoxide radicals that efficiently abstract hydrogen atoms and initiate chain scissions.^28,47,67,68^ Recent electron paramagnetic resonance (EPR) studies by Sayari confirmed a sharp increase in radical content during oxidation of PEI/silica sorbents in the presence of Fe.^34^ In contrast, Ni^2+^ is much less reactive under these conditions due to its sluggish redox kinetics and the less favorable Ni^3+^/Ni^2+^ redox couple, resulting in minimal radical formation and slower degradation.

Although oxidation experiments were intentionally performed in dry, CO_2_-free air to isolate the role of metals without the confounding effects of water-mediated speciation, prior studies have shown that both H_2_O and CO_2_ influence amine degradation kinetics and speciation. Water can enhance polymer mobility, modify oxygen transport, and alter hydroperoxide formation and decomposition pathways, as well as metal hydrolysis and coordination environments.^15^ CO_2_ exposure can further affect amine availability, carbamate chemistry, and radical propagation mechanisms.^14,62^ Under practical DAC conditions, these factors may quantitatively modify degradation rates. However, isolating metal effects under controlled dry conditions enables identification of intrinsic metal-dependent susceptibility, which provides a necessary mechanistic baseline upon which more complex humid and cyclic scenarios can be interpreted.

To further explore metal speciation and oxidation state, UV–vis diffuse reflectance spectroscopy was conducted on fresh and oxidized samples of control and metal-contaminated sorbents containing Cu, Fe, and Ni ions (Figure 5). The absorbance spectra were converted from reflectance (% *R*) data (Figure S9) using log­(1/*R*). The fresh Cu-containing sorbent shows a broad absorption band centered around 600 nm, characteristic of d–d transitions in Cu^2+^ complexes, likely arising from coordination to nitrogen ligands such as primary and secondary amines.^69,70^ Upon oxidation, this band becomes more intense and broader, indicating changes in the Cu coordination environment and potential oxidation state shifts. The fresh Fe-containing sample exhibits a relatively weak and broad feature between 300 and 450 nm, consistent with Fe^2+^ species. After oxidation, the absorbance in this region increases significantly, suggesting the formation of Fe^3+^ species via ligand-to-metal charge transfer (LMCT) transitions. This spectral evolution suggests a partial oxidation of Fe under dry aging conditions. The Ni-contaminated fresh sorbent displays a broad absorption from 300 to 700 nm, characteristic of octahedral Ni^2+^ complexes. Oxidation leads to a slight increase in overall absorbance, but no distinct new bands emerge, reflecting the lower redox activity of Ni compared to Cu and Fe. These observations suggest that metal contamination not only perturbs the electronic environment of PEI but also undergoes further transformation under oxidative conditions, potentially influencing the degradation pathway and kinetics.

**Figure 5:**
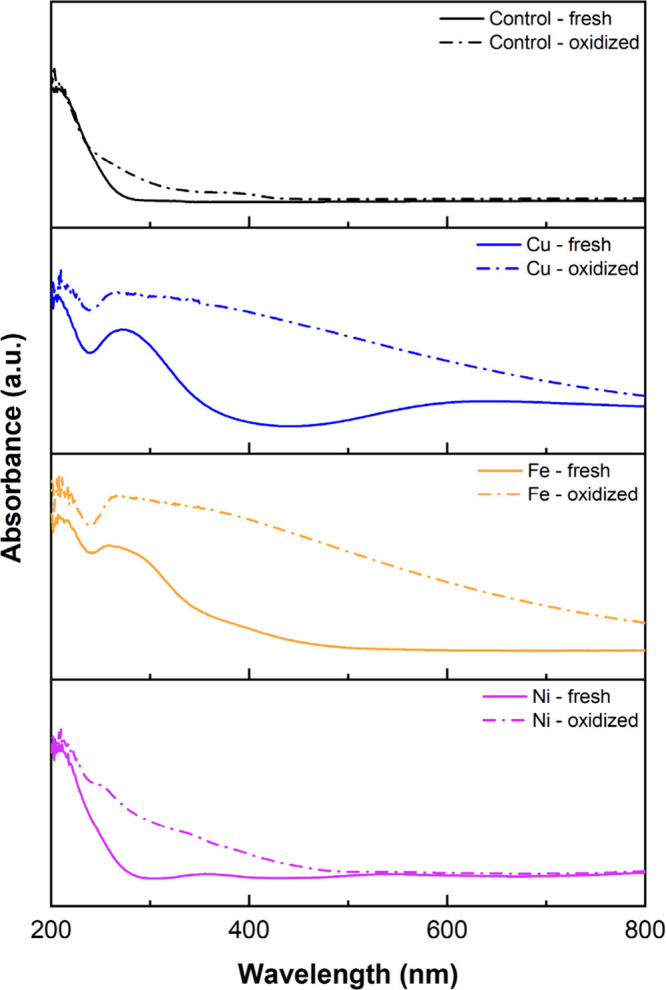
UV–vis diffuse reflectance spectra of control and metal-contaminated PEI sorbents (Cu, Fe, and Ni) in the fresh and oxidized states. Original reflectance data (% *R*; Figure S9) were converted to absorbance via log­(1/*R*).

In summary, the present work systematically evaluates the influence of transition metal contaminants on amine-functionalized solid sorbents for DAC across multiple contamination pathways (amine, support, and environmental deposition) at controlled metal loadings. By mapping the effects of Cu, Fe, and Ni across these pathways, this work establishes a mechanistic foundation that can guide subsequent studies under more realistic DAC operating conditions. The results highlight that transition metal ions, particularly Cu and Fe, severely compromise the long-term stability of PEI, the most widely studied polyamine sorbent for DAC. Even at ppm levels, these metals reduce CO_2_ capture capacity by up to 90–100% after a short oxidation period, primarily by coordinating with amines and catalyzing radical-driven C–N and C–C bond cleavage under oxidizing conditions. *In situ* DRIFTS experiments confirm that metals accelerate the formation of nonbasic oxidation products, such as carbonyls and imines, thereby deactivating sorbent sites. While different contamination pathways affected the initial CO_2_ performance of fresh sorbents, aged sorbents ultimately converged to similar low uptake levels, though degradation kinetics may differ depending on metal location and accessibility.

Most existing DAC deployment efforts to improve the oxidative resistance of sorbents do not account for environmental- or manufacturing-derived metal impurities. Our results emphasize the need to control metal content in sorbent synthesis and in the process air and water streams that contact the sorbent during operation. In practical systems, upstream filtration of incoming air streams to remove particulate-bound metals and the use of corrosion-resistant materials or appropriately conditioned boiler water could help limit the introduction of transition metals into DAC units. Beyond these process-level considerations, several stabilization strategies reported in the broader amine-oxidation literature may offer routes to improving the robustness of PEI-based sorbents. Chelating agents such as phosphate additives,^33^ when presupported on silica, can immobilize pre-existing metal impurities present in commercial amines or supports (modes 1 and 2). However, their efficacy is likely limited for ongoing environmental deposition (mode 3), because chelators are embedded within the support and possess finite binding capacity. Other potential stabilization strategies include chemical modification of amine groups to reduce oxidative susceptibility (e.g., epoxide functionalized),^33,34,71,72^ incorporation of hydrogen-bonding polymers such as polyethylene glycol,^55,73^ or poly­(vinyl alcohol)^34,55^ that alter the local microenvironment of the amines, and the use of inherently more oxidation-resistant amine structures such as poly­(propylenimine),^39,74^ and the incorporation of radical-scavenging additives designed to intercept reactive oxygen species and suppress chain propagation reactions.^75^ Each of these approaches also carries trade-offs, as added stabilizing phases can reduce pore volume and amine loading, whereas modified or alternative amine chemistries may decrease CO_2_ capacity or increase material cost. These strategies represent promising directions for future sorbent development but fall outside the diagnostic scope of the present mechanistic investigation. Finally, mode 3 contamination (environmental metal exposure) introduces a realistic framework for studying atmospheric contamination effects, setting the stage for broader investigations into other airborne species that may influence sorbent aging under field-relevant conditions.

Metal-accelerated degradation also carries broader environmental implications. Faster loss of sorbent performance shortens sorbent lifetimes, increasing materials and energy demand for DAC operation.^76^ In addition, such pathways can promote the formation of volatile nitrogen-containing species (e.g., ammonia and fragmented amines),^15,16,18^ which may participate in atmospheric particle formation and reactive nitrogen chemistry, impacting human health.^77,78^ These considerations underline the importance of limiting metal contamination to ensure both sorbent durability and environmentally responsible DAC operation.

Key limitations of this study include the use of dry and CO_2_-free conditions during accelerated aging, which may not fully capture the synergistic effects of humidity or ambient CO_2_ present in field DAC operation.^14^ All CO_2_ adsorption tests were also performed under dry conditions. Additionally, SBA-15 was chosen as a model support for its well-defined pore structure and low impurity content, but other supports (e.g., γ-Al_2_O_3_) may interact differently with amines and metal ions. Finally, oxidation experiments were conducted at 120 °C to accelerate aging kinetics, a temperature higher than typical DAC regeneration conditions (<100 °C), and thus may overestimate degradation rates.

## Supplementary Material



## Notes

Additional experimental data and characterization, including nitrogen sorption, SEM/TEM imaging, textural properties, ICP–MS metal quantification, elemental analysis and XPS, CO_2_ uptake measurements under various aging temperatures, DRIFTS spectra (fresh and *in situ*), UV–vis spectra, and a conceptual schematic of hypothesized metal-catalyzed PEI degradation pathways (PDF)
