# Supplementary material for: Influence of Transition Metal Ion Contaminants on the Performance of Amine-Based Solid Sorbents in Direct Air Capture
Source: Environ Sci Technol. 2026 May 21;60(21):14912–23. doi: 10.1021/acs.est.5c11392 (PMC13235560; doi:10.1021/acs.est.5c11392)
Supplement: Supplementary file 1 [file es5c11392_si_001.pdf]

Supporting Information

for

Influence of Transition Metal Ion Contaminants on Performance of Amine-Based Solid Sorbents in Direct Air Capture

Botagoz Kuspangaliyeva,[1] Ryan P. Lively,[1,*] Christopher W. Jones[1,*]

[1]School of Chemical & Biomolecular Engineering, Georgia Institute of Technology, Atlanta, GA 30332, United States

* correspondence: ryan.lively@chbe.gatech.edu, cjones@chbe.gatech.edu

Contents of this file

Supplementary Figure S1 to S9, Pages S2-S15;

Supplementary Table S1 to S5, Pages S3-S9;

References, Page S16.

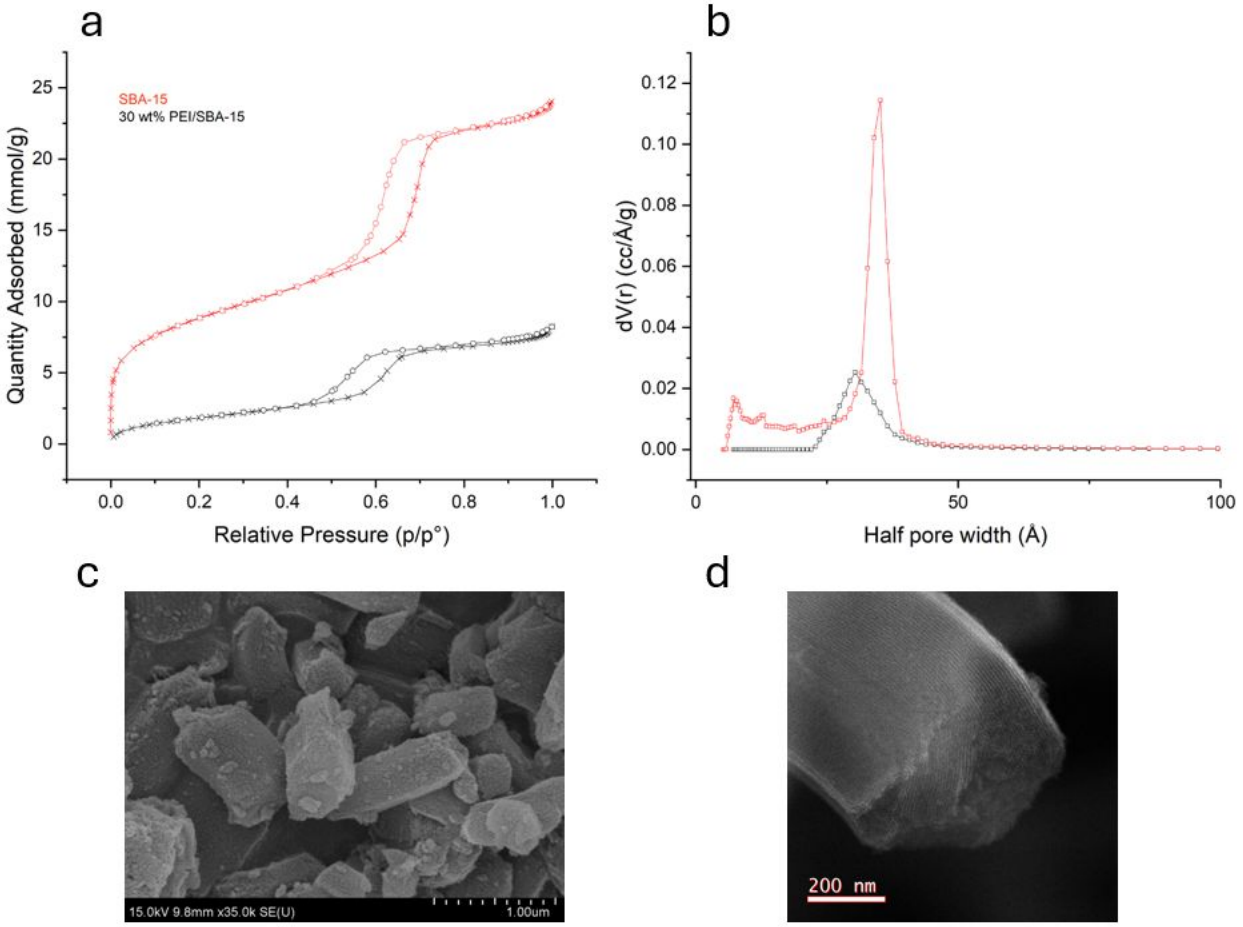

Figure S1. (a) Nitrogen physisorption isotherms for bare SBA-15 and PEI/SBA-15 composite sorbent. Open and closed symbols indicate desorption and adsorption steps of the isotherms, respectively. (b) Pore size distributions calculated via the NLDFT method. (c, d) Representative SEM and TEM images of the bare SBA-15 support.

Table S1. Summary of the textural properties of support, control, and metal-contaminated sorbents.

Samples	BET surface area (m^2/g)	Pore volume (cm^3/g)	Pore fill fraction (%)	Pore width (nm)
SBA-15	710	0.79	-	7
PEI/SBA-15	160	0.26	67	6
PEI:Cu 1:1	150	0.25	68	6
PEI:Fe 1:1	160	0.25	67	6
PEI:Ni 1:1	150	0.24	68	6

Table S2. CO_2 uptake and amine efficiency values corresponding to Figure 2.

**Metal**	**PEI:Metal (molar ratio)**	**Fresh (mean ± half-range, n = 2)**		**Oxidized (mean ± half-range, n = 2)**	
		**CO_2 uptake ($mmol_{CO2}/g_{PEI}$)**	**Amine efficiency (mol CO_2/mol N)**	**CO_2 uptake ($mmol_{CO2}/g_{PEI}$)**	**Amine efficiency (mol CO_2/mol N)**
Control	-	2.41 ± 0.02	0.10 ± 0.00	1.74 ± 0.14	0.07 ± 0.01
Cu	10,000:1	2.17 ± 0.01	0.09 ± 0.00	1.52 ± 0.01	0.07 ± 0.00
	1,000:1	2.18 ± 0.05	0.09 ± 0.00	0.61 ± 0.10	0.03 ± 0.00
	100:1	2.18 ± 0.08	0.09 ± 0.00	0.02 ± 0.01	0.00 ± 0.00
	10:1	2.13 ± 0.04	0.09 ± 0.00	0.00 ± 0.01	0.00 ± 0.00
	1:1	1.27 ± 0.09	0.05 ± 0.00	-0.02 ± 0.03	0.00 ± 0.00
Fe	10,000:1	2.33 ± 0.10	0.10 ± 0.00	1.67 ± 0.04	0.07 ± 0.00
	1,000:1	2.34 ± 0.05	0.10 ± 0.00	1.2 ± 0.10	0.05 ± 0.01
	100:1	2.26 ± 0.02	0.10 ± 0.00	0.12 ± 0.01	0.01 ± 0.00
	10:1	2.26 ± 0.03	0.10 ± 0.00	-0.02 ± 0.02	0.00 ± 0.00
	1:1	0.67 ± 0.05	0.03 ± 0.00	0.00 ± 0.01	0.00 ± 0.00
Ni	10,000:1	2.26 ± 0.10	0.10 ± 0.00	1.63 ± 0.02	0.07 ± 0.00
	1,000:1	2.31 ± 0.12	0.09 ± 0.01	1.58 ± 0.04	0.07 ± 0.00
	100:1	2.25 ± 0.11	0.10 ± 0.00	1.25 ± 0.03	0.05 ± 0.00
	10:1	2.12 ± 0.05	0.09 ± 0.00	0.77 ± 0.05	0.03 ± 0.00
	1:1	1.41 ± 0.01	0.06 ± 0.00	0.68 ± 0.08	0.00 ± 0.00

* Values are reported as mean ± half-range, where half-range = (max–min)/2 of two independently prepared samples (n = 2).

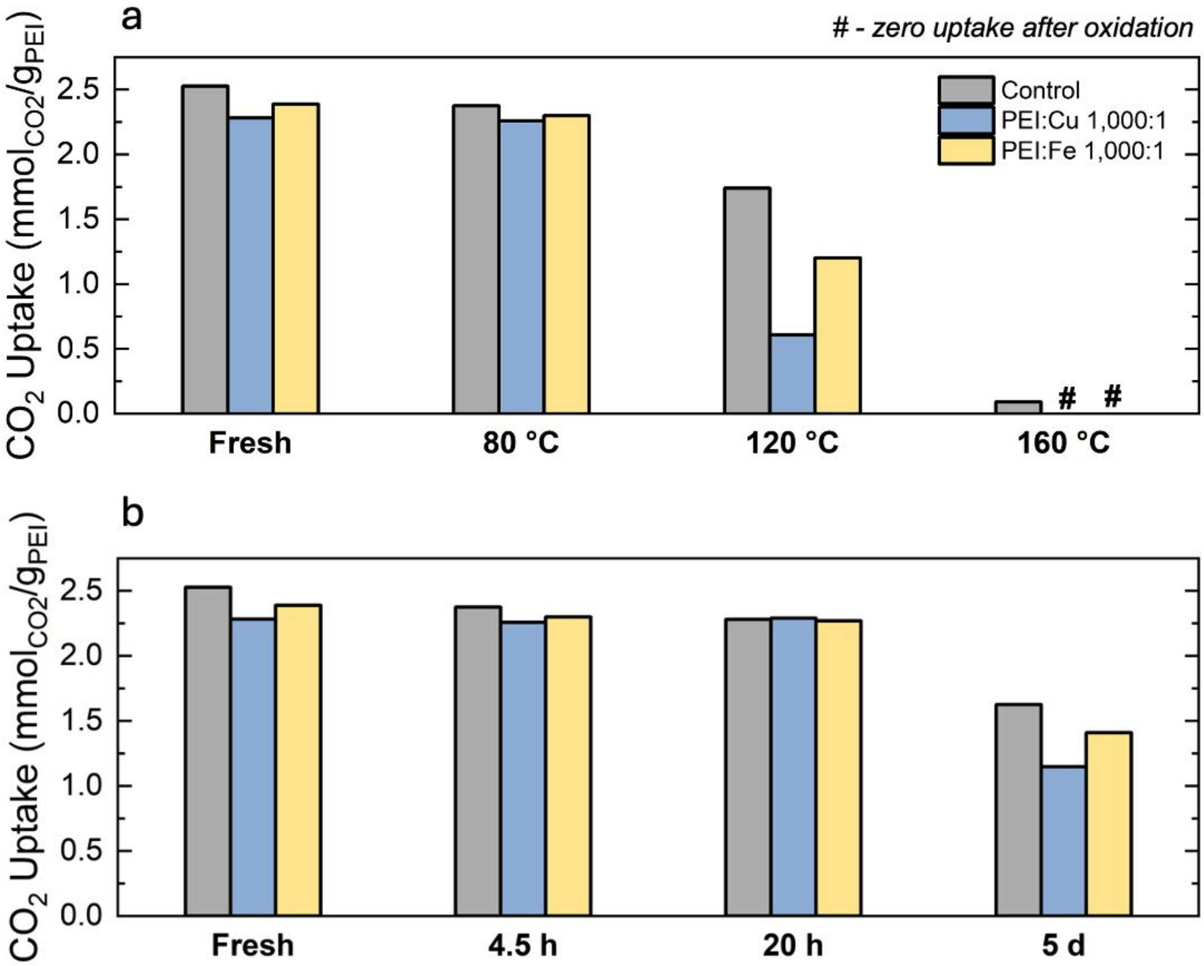

Figure S2. CO_2 uptake of control, Cu-, and Fe-contaminated sorbents (PEI:metal = 1,000:1 molar ratio) after oxidative aging under 21% O_2 in N_2. (a) Temperature-dependent aging for 4.5 h at 80, 120, and 160 °C. (b) Time-dependent aging at 80 °C (4.5 h, 20 h, and 5 d), illustrating preservation of relative metal-dependent trends under mild regeneration conditions. Measurements were performed using dry 400 ppm CO2 at 30 °C.

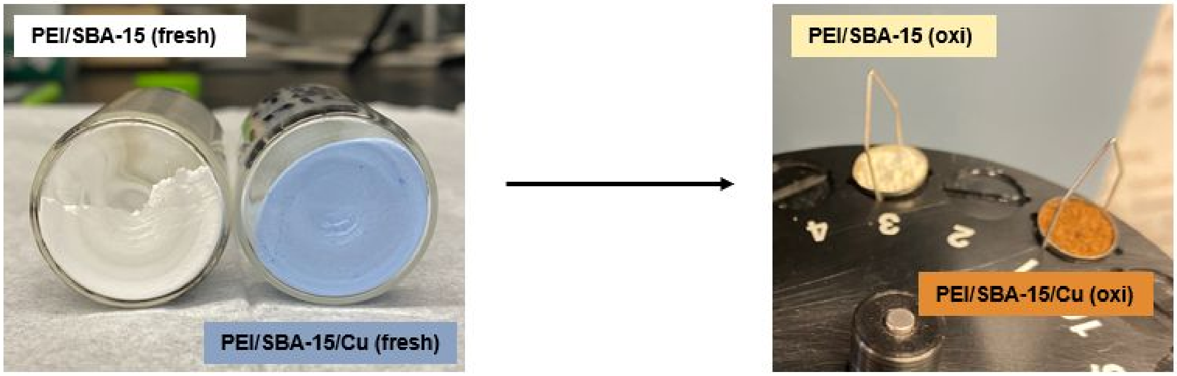

Figure S3. Color changes observed in fresh (left) and oxidized (right) control and Cu-contaminated sorbents.

Table S3. Transition metal content determined by ICP-MS in the synthesized support, commercial PEI, and metal-contaminated PEI samples (Mode 1).

Sample	Metal Concentration
SBA-15	0.2 ppm Cu<br>0.9 ppm Fe<br>0.1 ppm Ni
PEI	< 0.1 ppm Cu<br>< 0.5 ppm Fe<br>< 0.1 ppm Ni
PEI:Cu 1:1	1.83 % Cu
PEI:Fe 1:1	1.79 % Fe
PEI:Ni 1:1	1.60 % Ni
PEI:Cu 10,000:1	4 ppm Cu

Table S4. Metal-to-nitrogen molar ratios (bulk EA and surface XPS) and total metal concentrations determined by ICP-MS for selected contaminated fresh sorbents at PEI:Metal 1:1 molar ratio.

Mode	Contaminated Metal Sample	Metal/Nitrogen Molar Ratio*		Metal Concentration
		Elemental Analysis	XPS	
Mode 1	Cu	0.05	0.05	1.8 %
	Fe	0.05	0.09	1.8 %
	Ni	0.05	0.05	1.6 %
Mode 2.1	Cu	0.05	0.03	1.8 %
	Fe	0.04	0.09	1.5 %
Mode 2.2	Fe	0.05	0	1.7 %
Mode 3.1**	Cu	2.0×10^{-3}	0	792 ppm
	Fe	3.0×10^{-4}	0	112 ppm
	Ni	1.0×10^{-3}	0	467 ppm
Mode 3.2	Fe	0.05	0.11	1.7 %

* EA ≈ XPS → metal uniformly distributed
EA >> XPS → metal buried
EA << XPS → metal enriched at the surface

** Mode 3.1: final metal loading is lower than expected due to aerosol loss from the system

Table S5. Metal-to-silica molar ratios measured by XPS for selected contaminated sorbents at PEI:Metal 1:1 molar ratio.

Mode	Contaminated Metal Sample	Metal/Silica Molar Ratio (XPS)*	
		Fresh	Oxidized
Mode 1	Cu	0.02	0.02
	Fe	0.05	0
	Ni	0.03	0
Mode 2.1	Cu	0.02	0.02
	Fe	0.05	0
Mode 2.2	Cu	0	0.02
	Fe	0	0.02
	Ni	0	0
Mode 3.2	Cu	0.02	0.02
	Fe	0.08	0

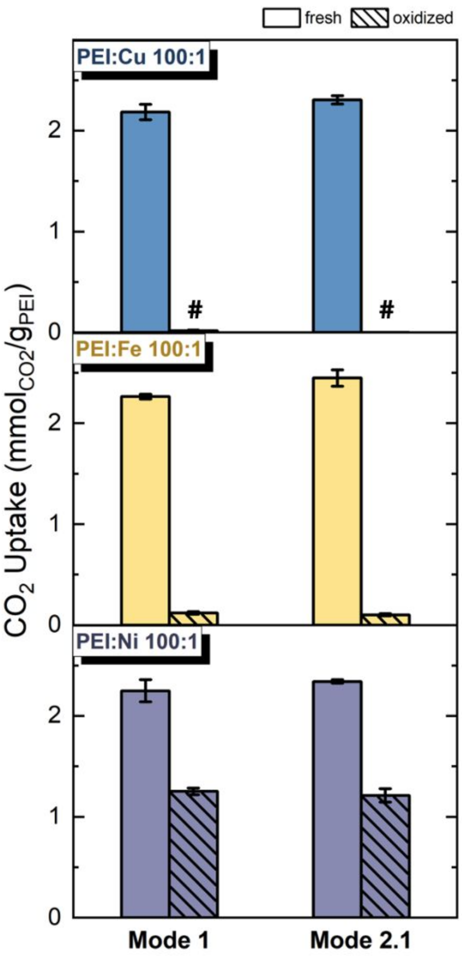

Figure S4. CO_2 uptake capacities of the sorbents contaminated with Cu, Fe, or Ni through two pathways, each prepared at a PEI:metal molar ratio of 100:1. Contamination pathways include: Mode 1 – metal incorporated in PEI; Mode 2.1 – metal mixed with support. Adsorption measurements were conducted before and after oxidative aging under 21% O_2 in N_2 at 120 °C for 4.5 h, using dry 400 ppm CO_2 at 30 °C. The # symbol marks samples with zero residual capacity. N = 2 independent samples were prepared and tested. Data represent the average, and error bars indicate the range.

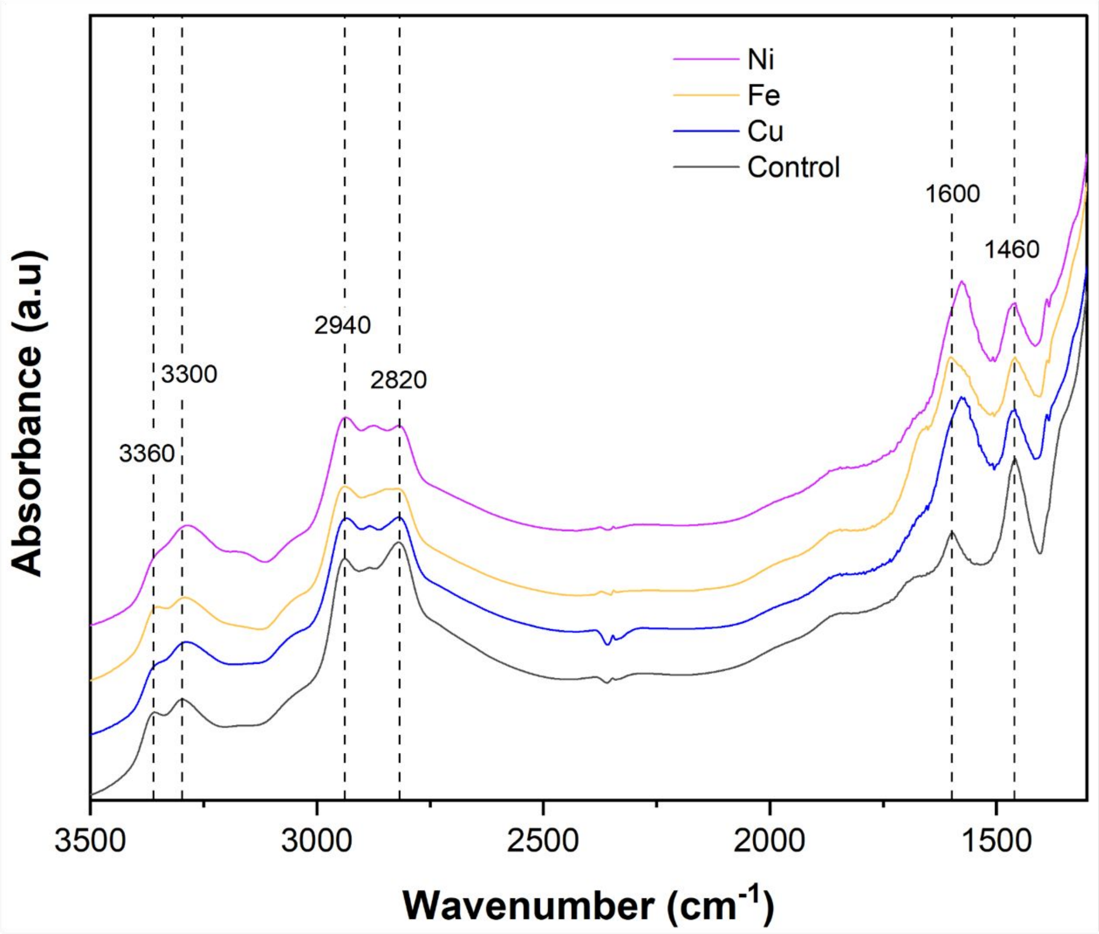

Figure S5. DRIFTS spectra of control and metal-contaminated sorbents (Cu, Fe, Ni) prepared via Mode 1 (metal-contaminated PEI). Spectra were collected after sample activation. Metal ions were introduced at a PEI:metal molar ratio of 1:1.

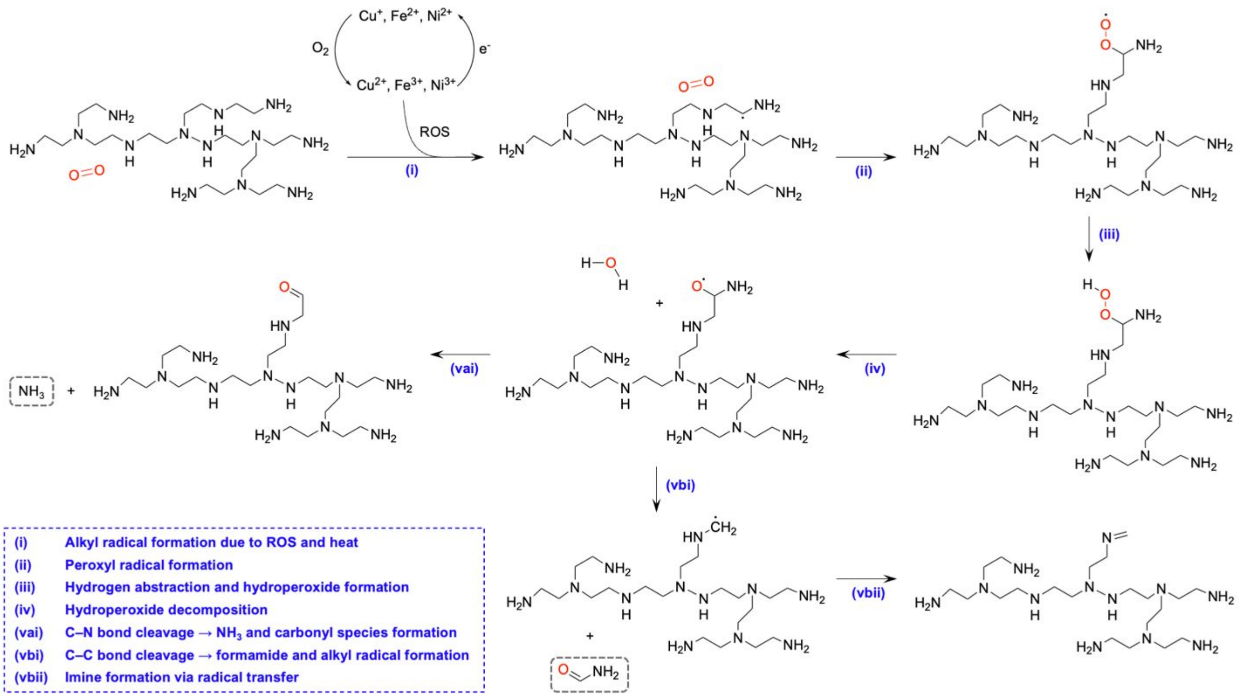

Figure S6. Conceptual schematic of hypothesized PEI oxidative degradation pathways in the presence of transition metal contaminants. Transition metal redox cycling (M^n/M^{n+1}) in the presence of O_2 may generate reactive oxygen species (ROS) that initiate alkyl radical formation. These radicals then feed into the suggested autoxidation pathways of branched PEI. The integrated C–N and C–C cleavage routes shown here are adapted from the proposed mechanisms of Carneiro et al.[1] and Li et al.,[2] respectively. Numbered steps (i-v) correspond to the key radical propagation and decomposition events.

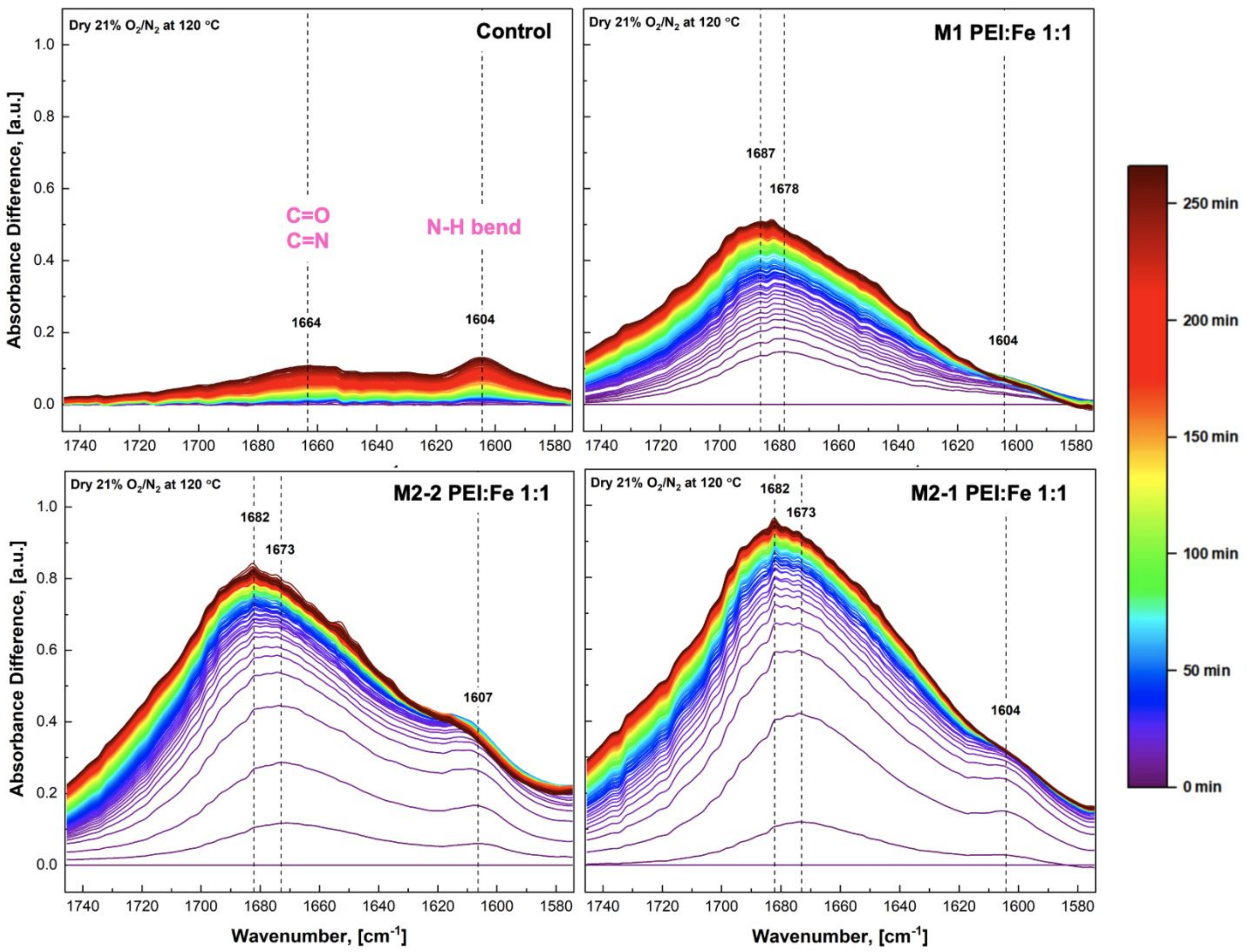

Figure S7. Difference DRIFTS spectra collected during in situ oxidative aging of (a) control and sorbents contaminated with Fe through (b) PEI (Mode 1), (c) calcined metal + support (Mode 2-2), (d) uncalcined metal + support (Mode 2-1) under 21% O_2 in N_2 at 120 °C for 4.5 h. All contaminated samples were prepared at a PEI:Fe molar ratio of 1:1.

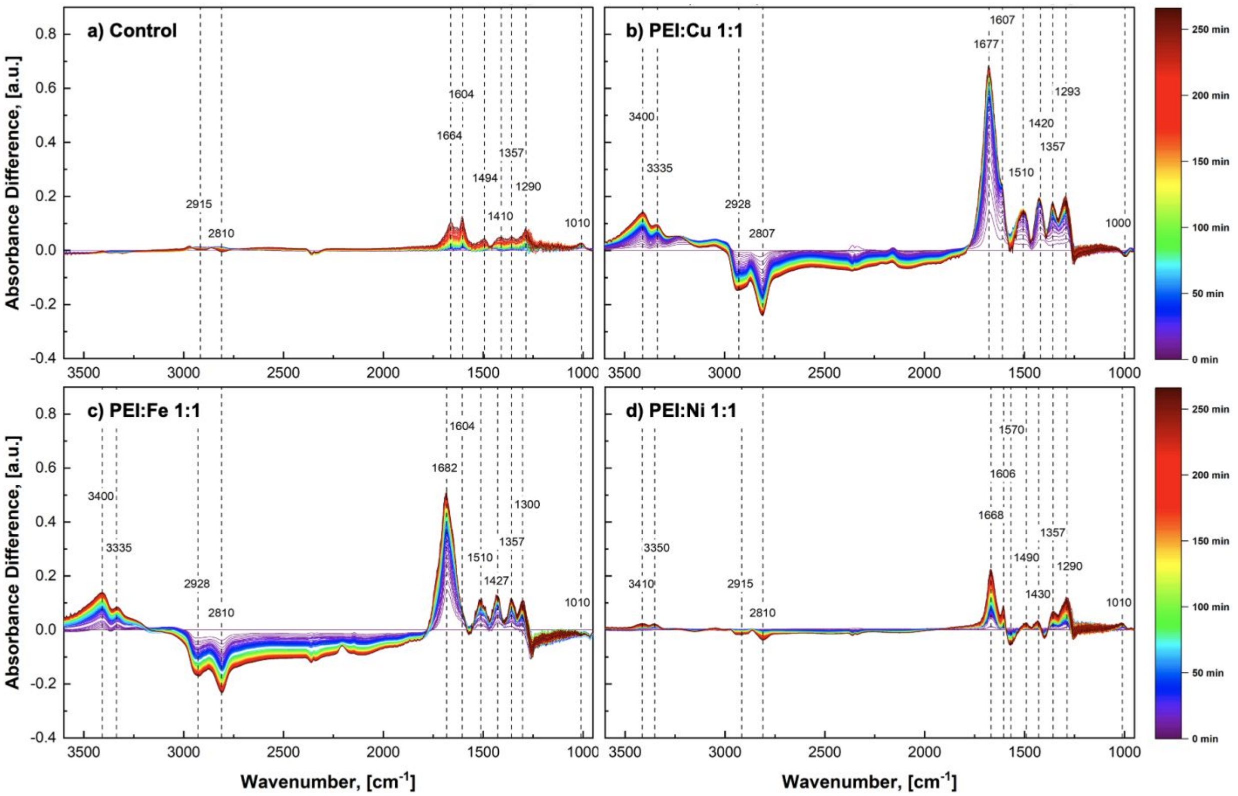

Figure S8. Difference DRIFTS full spectra collected during in situ oxidative aging of (a) control and (b-d) metal-contaminated PEI sorbents (Cu, Fe, Ni) under 21% O_2 in N_2 at 120 °C for 4.5 h. All contaminated samples were prepared at a PEI:metal molar ratio of 1:1.

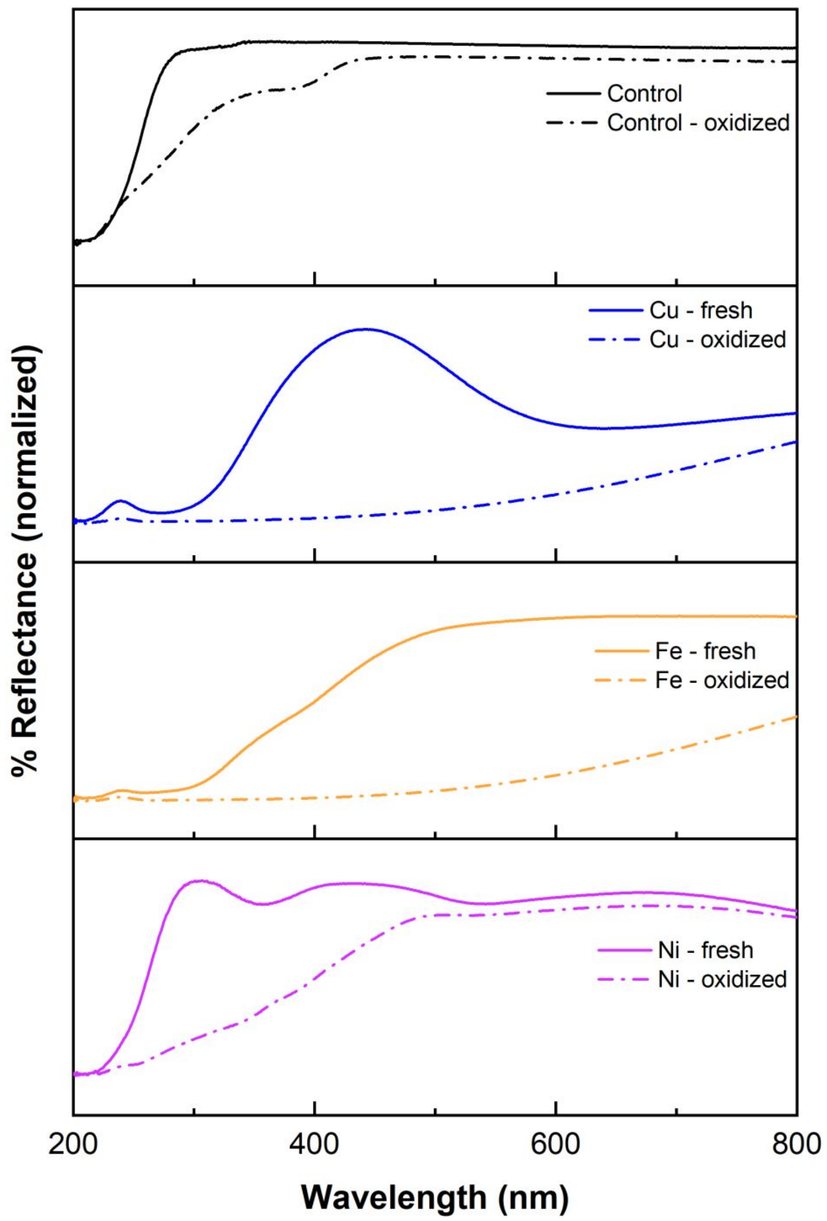

Figure S9. Normalized diffuse reflectance (%R) spectra of control and metal-contaminated PEI sorbents (Cu, Fe, Ni) in the fresh and oxidized states.

References

(1) Carneiro, J. S. A.; Innocenti, G.; Moon, H. J.; Guta, Y.; Proaño, L.; Sievers, C.; Sakwa-Novak, M. A.; Ping, E. W.; Jones, C. W. Insights into the Oxidative Degradation Mechanism of Solid Amine Sorbents for CO_2 Capture from Air: Roles of Atmospheric Water. *Angew. Chem. Int. Ed.* **2023**, *62* (24), e202302887. https://doi.org/10.1002/anie.202302887.

(2) Li, S.; Guta, Y.; Calegari Andrade, M. F.; Hunter-Sellars, E.; Maiti, A.; Varni, A. J.; Tang, P.; Sievers, C.; Pang, S. H.; Jones, C. W. Competing Kinetic Consequences of CO_2 on the Oxidative Degradation of Branched Poly(Ethylenimine). *J. Am. Chem. Soc.* **2024**, 146, 41, 28201–28213. jacs.4c08126. https://doi.org/10.1021/jacs.4c08126.
